# Pharma Learns to Share as Their AI Demands More Data

**DOI:** 10.1021/acscentsci.6c00643

**Published:** 2026-04-20

**Authors:** Eva Amsen

## Abstract

Companies aim to improve drug discovery by training AI on one another’s data and generating large, open data sets.

## Graphical abstract

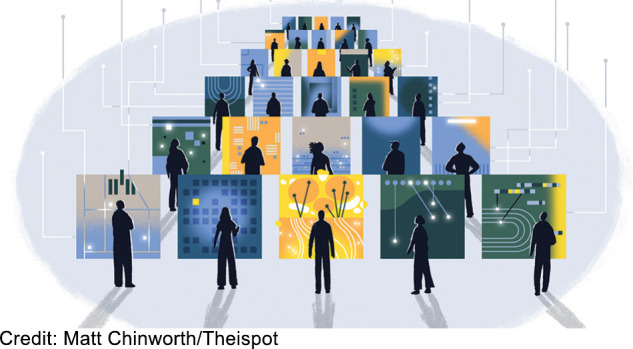

After artificial intelligence tools like AlphaFold learned to predict protein structures, using machine learning in drug development seemed like a logical next step. But it takes more than protein structures alone to narrow down new drug candidates. Drug developers also need to understand how the drug will interact with a protein, as well as the rest of the body, before deciding which drug candidates to focus on.

**Figure:**
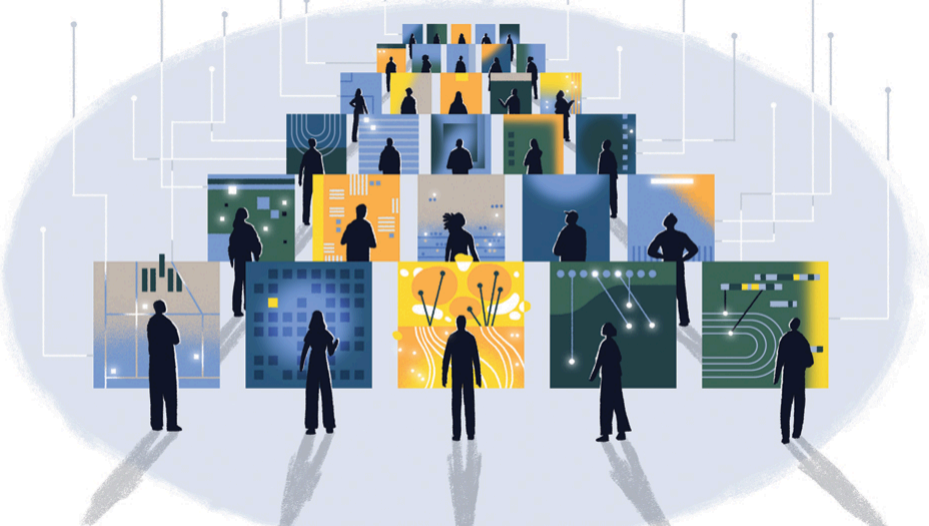
Credit: Matt Chinworth/Theispot.

In the past few years, researchers have developed machine learning tools to support these processes, but something is missing. There are “astonishingly little data” on drug-target interactions, according to Frank von Delft, a structural biologist at the University of Oxford and principal beamline scientist at Diamond Light Source, a particle accelerator facility. “All machine learning is completely dependent on how much data there are,” he says.

Some of the data that AI tools need for drug development predictions have never been systematically recorded, while others are kept in proprietary industry databases. But motivated by a joint goal to make machine learning work for drug development, pharmaceutical companies are collaborating with one another and with public consortia to close the data gap.

## The demand for better training data

In early stage drug development, computational modeling helps researchers narrow down which molecules should move on to further preclinical development. Machine learning can speed up and expand many of these early steps with tools that predict drug absorption, distribution, metabolism, excretion, and toxicology (ADMET), or by mapping drug-protein interactions according to their predicted binding affinities and 3D structures.

All these tools need training data. For example, tools that predict protein structures, like AlphaFold, were trained on the extensive archive of the Protein Data Bank (PDB). This resource currently holds around 250,000 protein structures which were added over several decades. But AI tool developers need more complex data than protein structures alone if they want to support drug development.

“There aren’t that many structures of proteins bound to small molecules,” says Mohammed AlQuraishi, assistant professor of systems biology at Columbia University. AlQuraishi’s team developed OpenFold, an open-source tool that predicts protein folding structures. Its latest version, OpenFold3, also models complexes of proteins with small molecules.

Drug developers’ outsized need to understand interactions between potential drugs and proteins is demonstrated by the broad usage of another open-source model, Boltz-2. This tool, which predicts structures and binding affinities for interactions between proteins and other molecules, has been downloaded over 1.2 million times, says Regina Barzilay, an electrical engineering and computer science professor at the Massachusetts Institute of Technology whose team developed Boltz. “It’s really very broadly used, and mostly by people who design drugs,” she says.

**Figure:**
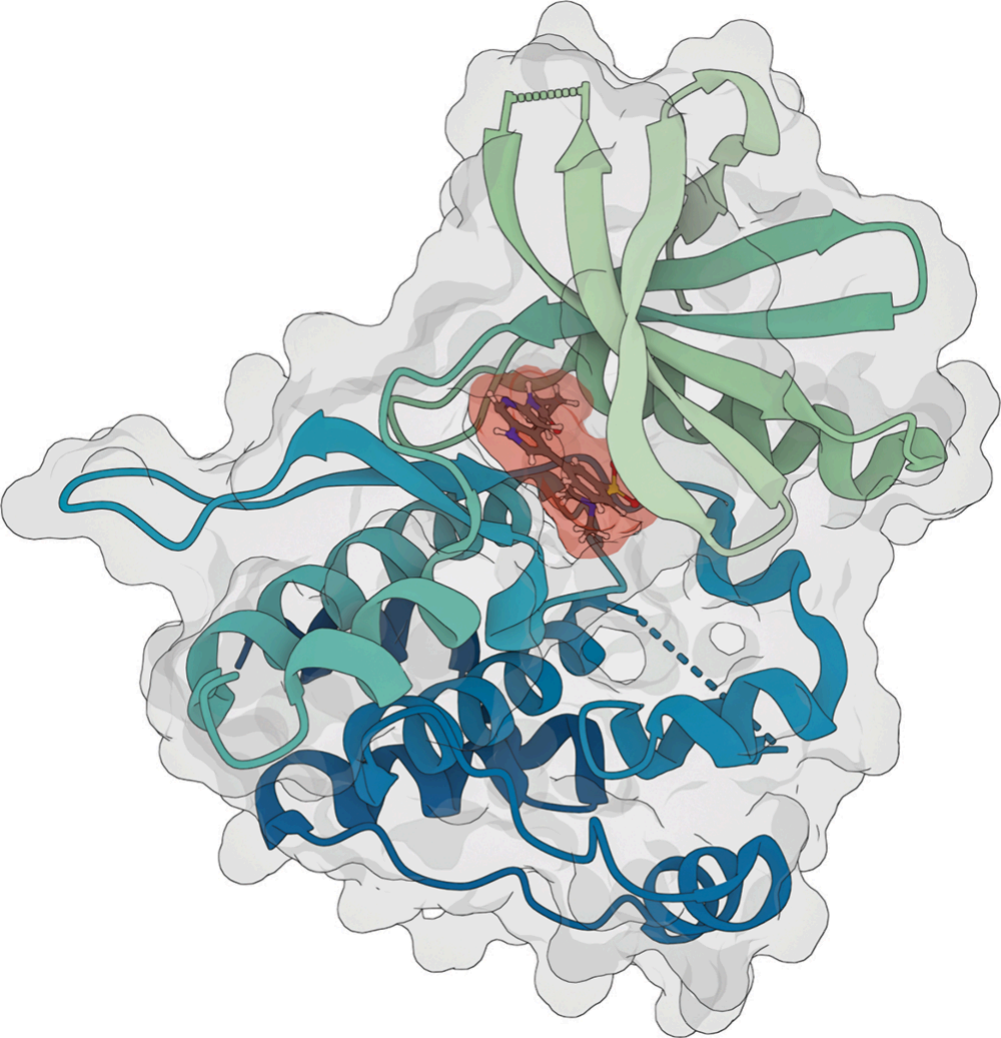
A predicted structure generated with Boltz-2 of a tyrosine kinase 2 protein bound to a small molecule (shaded orange) that could act as a selective inhibitor. Credit: MinGyu Choi/Massachusetts Institute of Technology.

Having more training data would greatly improve how tools like AlphaFold, OpenFold3, and Boltz-2 predict drug–protein interactions. Tools that model ADMET properties also rely on access to relevant drug data. But many of these data are not publicly available, as PDB data are. Instead, they are often siloed in proprietary pharmaceutical databases.

“Since the users of these tools are pharma companies, it’s in their benefit either to sponsor data creation or share data so that we can all benefit from it,” Barzilay says.

## Federated learning on existing data

One of the solutions to the shortage of training data has come from pharmaceutical companies themselves: training AI tools without having access to one another’s data. In this approach, called federated learning, participating organizations train a copy of the tool’s algorithm on their own data; they share only how their data updated the algorithm—not the data themselves.

Federated learning relies on collaboration among pharma companies. For example, Eli Lilly and Company lets other companies access some of its machine learning tools through its TuneLab platform. In return, TuneLab users are allowing Lilly to train the models on their data. “What we’re most excited about, of course, is improving the models themselves,” says Aliza Apple, global head of Lilly TuneLab. “That’s the entire premise of the project.”

Meanwhile, an initiative called FAITE (federated AI therapeutic engineering) is focused on biologic drugs, such as antibodies used for immunotherapy. These biologics come with their own challenges. “Biologic data are often generated under different experimental conditions, using varied assays and formats,” says Marti Head, associate vice president of research at Amgen, one of the companies taking part in FAITE. This “leads to fragmentation and limits the ability of models to perform well across programs or organizations,” she adds. FAITE is now running a pilot between five pharma companies to see if federated learning can offer a way for companies to securely train AI tools on their collective biologics data.

Projects like TuneLab and FAITE learned a lot from MELLODDY (machine learning ledger orchestration for drug discovery), a federated learning effort that ran from 2019 to 2022. The 10 pharma companies that took part in MELLODDY were the first to train AI tools together without revealing their data. “It was an experiment to understand the technology for federated learning,” says Ola Engkvist, a computational chemist at AstraZeneca who was involved in the MELLODDY project.

MELLODDY—which also involved academics and technology companies, including Nvidia—showed that federated learning was possible but wasn’t always fair. “If you put a lot of data in, you didn’t get much out. If you put a little bit of data in, you got more out,” says Andrew Buchanan, head of discovery at a biotech start-up that is still in stealth and a former principal scientist at AstraZeneca.

Likewise, federated learning initiatives need to ensure that the data each partner contributes are compatible in quality and reproducibility. “When you’re going to share data, you have to agree on a data standard,” Buchanan says.

To avoid imbalanced participation, TuneLab uses an interview process to vet potential partners, which includes an assessment of “the rigor behind their science and making sure that it’s a good fit overall for their scientific needs,” Apple says. At its launch last year, TuneLab received more than 600 applications from companies wanting access to its AI tools, which will initially focus on predicting ADMET properties or the likelihood that a drug candidate can be manufactured. Only about 50 applicants have made the cut so far.

Even with clear rules about who takes part and which data they share, an issue remains: the existing data are limited. Most data collected in the drug discovery process cover only a subset of molecules relevant to known diseases. AI algorithms also need to learn about molecules that aren’t well studied and about the experiments that generated negative data. “It’s diversity of the data: that is a bottleneck,” says Engkvist at AstraZeneca.

## Generating new data

“Instead of doing more complex federated learning, maybe it’s better to get together to generate data and put it in the public domain,” Engkvist says. His company is part of a recently launched project, called Ligand-AI, that aims to do just that.

Led by Pfizer and the Structural Genomics Consortium (SGC), Ligand-AI is a collaboration of several pharma companies, universities, hospitals, and tech firms that are producing data on how proteins interact with billions of small molecules. These data are generated specifically to train AI tools.

“We generate new data going forward, openly as a public good,” says Aled Edwards, CEO of the SGC and professor of molecular genetics at the University of Toronto. This means that any developer of AI tools can use these data—akin to how protein folding predictors used public PDB data.

A similar project for open-data generation is OpenBind, led by Diamond Light Source, which houses a specialized particle accelerator known as a synchrotron. “Our basic premise is that we should measure the data that’s most directly useful,” von Delft says. To this end, OpenBind aims to use the synchrotron’s crystallography facilities to solve structures of proteins bound to small molecules and measure the binding affinities of these interactions. Once the first set of structures is made public, OpenBind hopes to encourage other facilities to join and eventually to solve half a million structures together.

**Figure:**
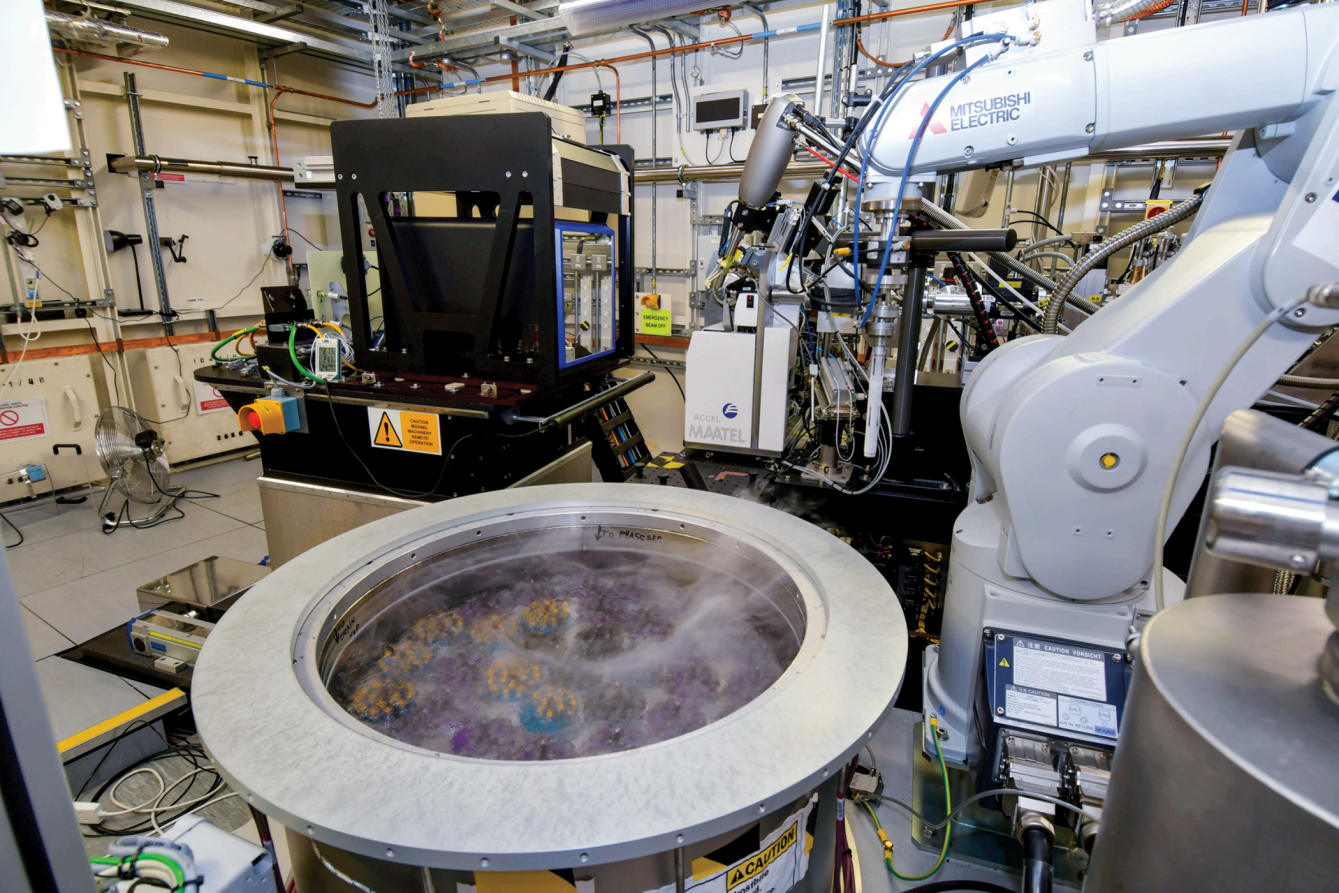
Crystals of proteins bound to small molecules are kept in liquid nitrogen before being placed in the beamline of the Diamond Light Source synchrotron. Protein-ligand structures solved here with X-ray crystallography will be collected as part of the OpenBind project to train AI tools. Credit: Diamond Light Source.

The real value of such large, open datasets won’t be apparent until the data are used to train AI tools. “It’s a tight coupling between the data and the modeling,” says AlQuraishi at OpenFold. “The modeling informs what data we need to acquire, and the data improves the quality of the models that can then feed the next cycle.” OpenFold already has an agreement to test its models on new structure data from OpenBind, but other tools will have similar opportunities since the data are unrestricted.

One challenge with large-scale data generation is storage. Ligand-AI plans to host its data in its own database, which was created with AI tool developers in mind. Unlike traditional databases, this one doesn’t require developers to download enormous data sets just to test their tools. Instead, it offers an interactive environment in which users can also run their AI tools. “You can upload your models and play with it,” Edwards says.

Meanwhile, OpenBind structures will find a home in the PDB. “We’re working with them to automate these large-scale depositions,” says Jennifer Fleming, PDB Europe coordinator at the European Molecular Biology Laboratory’s European Bioinformatics Institute (EMBL-EBI). The new structures will greatly expand the data on ligand-protein pairs that the PDB holds.

But the reach is even further. EMBL-EBI also hosts predicted structures from AlphaFold and other relevant databases, which connect to the PDB. For instance, if someone searches a protein on EMBL-EBI’s AlphaFold protein structure database, it shows the AlphaFold structure prediction as well as any known structures in the PDB. “I really want us to create a hub for structural biology,” Fleming says.

## Future of AI in drug development

Ultimately, both data generation projects and federated learning have the same goal: to improve AI tools for drug development. This shared mission has inspired several collaborations between developers of open-access tools and coordinators of federated learning initiatives.

For example, OpenFold is working with the AI Structural Biology Network, a federated initiative between a number of pharma companies. Without revealing their data, the companies will train OpenFold3 so that the public tool improves for everyone. This will mean more training data for OpenFold3, as well as “more relevant data, because this data is on drug or drug-like compounds,” AlQuraishi says.

Von Delft is also keen for OpenBind to partner with pharmaceutical companies. “They’re actively doing drug discovery,” so they can feed back whether the collected data are indeed improving discovery tools, he says. That kind of input is critical to determine whether new or existing AI tools are making a difference.

While AI tools can get better at selecting drug candidates, they probably won’t significantly accelerate the overall drug development process. “On average, the invention of a medicine takes 10–20 years from the initial discovery of the target,” Edwards says. “We may accelerate the first year to 6 months.”

But what these tools will be able to do is free up some time for the researchers involved in these early computational stages. Barzilay points out that they can encourage more creative ideas about producing new leads. “They can send you into more promising parts of the space, which you can experimentally validate afterwards.”

The recent surge of data generation and federated learning shows that pharma companies and public institutions have high hopes for AI in drug development. But that journey is just beginning. No AI-designed drugs have made it to market yet; the first ones are still going through clinical trials. “AI is a marathon,” Engkvist says. “It’s not a sprint.”

*Eva Amsen is a freelance contributor to* Chemical & Engineering News, *an independent news publication of the American Chemical Society.*

